# A Deep Learning–Based Approach for Prediction of Vancomycin Treatment Monitoring: Retrospective Study Among Patients With Critical Illness

**DOI:** 10.2196/45202

**Published:** 2024-03-08

**Authors:** Dohyun Kim, Hyun-Soo Choi, DongHoon Lee, Minkyu Kim, Yoon Kim, Seon-Sook Han, Yeonjeong Heo, Ju-Hee Park, Jinkyeong Park

**Affiliations:** 1 Department of Research and Development, ZIOVISION Co, Ltd Chuncheon Republic of Korea; 2 Department of Computer Science and Engineering Seoul National University of Science and Technology Seoul Republic of Korea; 3 Department of Computer Science and Engineering Kangwon National University Chuncheon Republic of Korea; 4 Department of Internal Medicine Kangwon National University Chuncheon Republic of Korea; 5 Department of Internal Medicine Dongguk University Ilsan Hospital Goyang Republic of Korea; 6 Department of Pulmonary, Allergy and Critical Care Medicine, School of Medicine Kyung Hee University Hospital at Gangdong Seoul Republic of Korea

**Keywords:** critically ill, deep learning, inflammation, machine learning, pharmacokinetic, therapeutic drug monitoring, vancomycin

## Abstract

**Background:**

Vancomycin pharmacokinetics are highly variable in patients with critical illnesses, and clinicians commonly use population pharmacokinetic (PPK) models based on a Bayesian approach to dose. However, these models are population-dependent, may only sometimes meet the needs of individual patients, and are only used by experienced clinicians as a reference for making treatment decisions. To assist real-world clinicians, we developed a deep learning–based decision-making system that predicts vancomycin therapeutic drug monitoring (TDM) levels in patients in intensive care unit.

**Objective:**

This study aimed to establish joint multilayer perceptron (JointMLP), a new deep-learning model for predicting vancomycin TDM levels, and compare its performance with the PPK models, extreme gradient boosting (XGBoost), and TabNet.

**Methods:**

We used a 977-case data set split into training and testing groups in a 9:1 ratio. We performed external validation of the model using 1429 cases from Kangwon National University Hospital and 2394 cases from the Medical Information Mart for Intensive Care–IV (MIMIC-IV). In addition, we performed 10-fold cross-validation on the internal training data set and calculated the 95% CIs using the metric. Finally, we evaluated the generalization ability of the JointMLP model using the MIMIC-IV data set.

**Results:**

Our JointMLP model outperformed other models in predicting vancomycin TDM levels in internal and external data sets. Compared to PPK, the JointMLP model improved predictive power by up to 31% (mean absolute error [MAE] 6.68 vs 5.11) on the internal data set and 81% (MAE 11.87 vs 6.56) on the external data set. In addition, the JointMLP model significantly outperforms XGBoost and TabNet, with a 13% (MAE 5.75 vs 5.11) and 14% (MAE 5.85 vs 5.11) improvement in predictive accuracy on the inner data set, respectively. On both the internal and external data sets, our JointMLP model performed well compared to XGBoost and TabNet, achieving prediction accuracy improvements of 34% and 14%, respectively. Additionally, our JointMLP model showed higher robustness to outlier data than the other models, as evidenced by its higher root mean squared error performance across all data sets. The mean errors and variances of the JointMLP model were close to zero and smaller than those of the PPK model in internal and external data sets.

**Conclusions:**

Our JointMLP approach can help optimize treatment outcomes in patients with critical illnesses in an intensive care unit setting, reducing side effects associated with suboptimal vancomycin administration. These include increased risk of bacterial resistance, extended hospital stays, and increased health care costs. In addition, the superior performance of our model compared to existing models highlights its potential to help real-world clinicians.

## Introduction

Vancomycin is frequently used for severe infections caused by gram-positive bacteria (including methicillin-resistant *Staphylococcus aureus*), such as pneumonia, skin and soft tissue infections, and other sepsis or septic shock in patients with critical illnesses. For vancomycin, because of the narrow therapeutic range and individual differences in pharmacokinetic parameters, therapeutic drug monitoring (TDM) is recommended to minimize toxicity and improve therapeutic efficacy. Although various pharmacokinetic indicators related to the antibacterial action of vancomycin have been suggested, the most recent guideline [1] recommends that a ratio of the area under the curve (AUC) over 24 hours to the minimum inhibitory concentration (MIC) of ≥400 should be the primary pharmacokinetic and pharmacodynamic (PK/PD) predictor of vancomycin activity [2]. However, in the real world, especially in intensive care units (ICUs), a more practical method of vancomycin monitoring is evaluating with trough concentrations since calculating the AUC requires multiple blood samples per patient [3-5].

There are different methods to determine the vancomycin AUC, such as the Bayesian approach or equation-based methodologies such as the trapezoidal model. The Bayesian approach uses population data to estimate individual patient pharmacokinetic parameters, called Bayesian priors. However, this method requires complex mathematical calculations to estimate the posterior distribution of model parameters and relies on certain assumptions, such as the normality of errors and data independence. Violating these assumptions can result in biased results, and using the Bayesian approach requires expensive commercial software programs [6,7]. On the other hand, equation-based approaches, like the trapezoidal model, do not require specialized software but need multiple blood sampling to achieve 2 steady-state levels. Since it is done under steady-state conditions, it cannot account for potential changes in AUC due to ongoing acute physiological changes. The pharmacokinetic profile of vancomycin exhibits linear pharmacokinetics over a range of therapeutic doses, is highly variable, especially in older people or people with critical illnesses, and is different for adults with normal renal function [8,9].

Population pharmacokinetic (PPK) models of vancomycin required to determine the AUC have very limited robustness beyond the specific population studied. The training and experience of the clinician or clinical pharmacist are still vital in achieving adequate vancomycin TDM, especially in patients with critical illnesses with high heterogeneity. A more multidimensional and consistent decision-making system is needed in such a clinical decision-making system because differences inevitably influence the results of individual clinicians’ abilities. Machine learning algorithms have emerged as a promising approach for improving decision-making on specific questions related to rich multidimensional data and for medical research and clinical care [10-12]. In previous studies, machine learning techniques such as decision trees [13] and extreme gradient boosting (XGBoost) [14,15] were used to make better predictions on vancomycin concentrations. In addition, various deep learning models have had great success in various fields [16], including clinical practice. These might help solve more complex clinical problems. In this study, we aimed to establish an ideal model by comparing and integrating methods that have been successful so far in the decision-making system that predicts the vancomycin TDM level in patients in the ICU.

## Methods

### Study Population

This retrospective study involved 2406 patients with critical illnesses admitted to medical ICUs, including 1 each at Dongguk University Ilsan Hospital (DUIH) and Kangwon National University Hospital (KNUH) between January 1, 2010, and February 28, 2022. We used the data collected from DUIH and KNUH as the internal and external validation data sets. The internal validation data set contained 977 patients, while the external validation data set contained 1429 patients.

Patients with critical illnesses (>18 years) with intravenous vancomycin treatment history and who had at least 1 test for vancomycin TDM were eligible for this study. Vancomycin TDM was investigated for trough-level information. If the TDM was checked multiple times, only the first TDM value was selected. However, in patients with normal renal function, if the interval between stopping vancomycin and readministering it was 2 weeks or more, it was considered an independent TDM value and used for analyses. Patients who received oral vancomycin or were aged 18 years or younger were excluded. The total serum vancomycin concentrations were determined using a fluorescence immunoassay (VANC3, Cobas c 702; Roche Diagnostics).

The data set was split into training and testing groups in a 9:1 ratio, with 879 and 98 cases representing the internal data set, respectively. For external validation, 1429 cases from KNUH were used. To evaluate the model’s generalization and the range of expected errors of the classifiers, 10-fold cross-validation was performed on the internal training data set, with a 9:1 ratio and 791 and 88 cases, respectively. The model was tested on both the internal and external test data sets for each fold and the metrics obtained were used to calculate the 95% CI over the 10 folds. The internal and external data sets remained constant across all folds. To evaluate the generalization ability of the joint multilayer perceptron (JointMLP) model, we used 2394 cases from the Medical Information Mart for Intensive Care–IV (MIMIC-IV) [17] as a US critical care database used for large-scale multi-institutional research for external validation.

### Experimental Model

**Figure 1:**
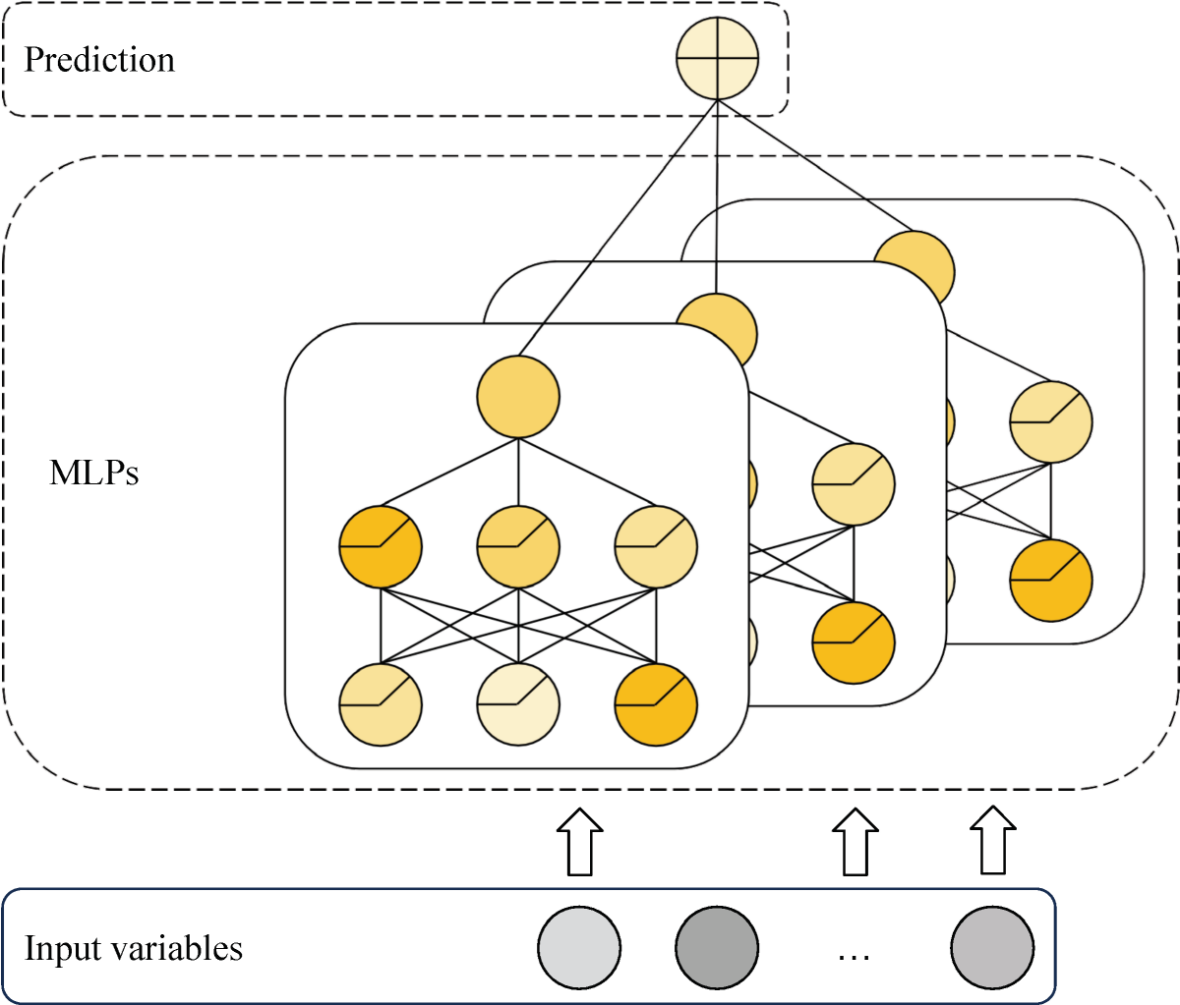
The architecture of joint multilayer perceptron (JointMLP). The JointMLP model was built with 100 different multilayer perceptrons (MLPs) consisting of 3 hidden layers with a hidden unit size of 32 and LeakyReLU as an activation function.

We proposed a JointMLP to predict the vancomycin TDM level in patients in the ICU. The JointMLP model is an ensemble model, meaning it combines the predictions of multiple individual MLPs (multilayer perceptrons) to generate a final prediction. The MLP is an artificial neural network. The MLP consists of multiple layers of interconnected nodes (also known as neurons) that perform mathematical operations on the input data to generate an output. In the case of the JointMLP model proposed for vancomycin TDM prediction, each MLP in the ensemble consists of 3 hidden layers with a hidden unit size of 32, and the LeakyReLU activation function is used to introduce nonlinearity into the model (Figure 1). Using an ensemble helps reduce the risk of overfitting and improves the accuracy and robustness of the model. In this case, the JointMLP model comprises 100 different MLPs, each with its own set of weights learned during the training process. To find the best set of hyperparameters for the JointMLP model, grid search is used, which systematically tests different combinations of hyperparameters to find the combination that yields the best performance on a validation data set. In this case, the tuned hyperparameters included the number of MLPs, the number of hidden layers, and the size of the hidden units. In a JointMLP model, the MLPs are trained independently in parallel. During training, each MLP in the ensemble receives a randomly selected subset of the training data and learns to make predictions based on that subset. The individual MLPs are then combined to generate a final prediction, typically by taking the average of their outputs or using a weighted combination. The MLPs are trained independently of each other and in parallel. Our JointMLP model is available on the websites [18] for predicting TDM levels and recommending the frequency and dose.

We compared 4 other models: the PPK model [19], XGBoost, TabNet, and 300-layer MLP. The PPK is a pharmacokinetic model used to assess the relationship between drug concentration and time in the body. XGBoost is an open-source library for decision tree–based gradient-boosting machine learning. The XGBoost model especially works well on tabular data learning. It is a vital model for distributed training or regularization. TabNet is a deep learning model specialized for tabular data set analysis. TabNet could be optimized by automatically transforming input variables and using additional validation data to select the required variables.

For XGBoost, the hyperparameters are eta (learning rate), gamma (minimum loss reduction required to make a further partition on a leaf node of the tree), and max_depth (maximum depth of a tree). The grid search was performed to find the best combination of these hyperparameters. The eta, gamma, and max depth values were 0.2, 0.001, and 6, respectively.

For TabNet, the hyperparameters are n_d (width of the decision prediction layer), n_a (width of the attention embedding for each mask), n_steps (number of steps in the architecture), gamma (coefficient for feature reusage in the masks), and verbose (verbosity for notebook plots). The grid search was performed to find the best combination of these hyperparameters. The values chosen for n_d, n_a, n_steps, gamma, and verbose were 8, 8, 3, 1.3, and 0, respectively.

The 300-layer MLP model was constructed to verify the performance of the JointMLP model. The hyperparameters for the MLP model were chosen to have the same depth as the JointMLP model, which is 100 horizontal MLPs times 3 hidden layers. The number of hidden layers was set to 300, and the number of hidden units was set to 32.

### Data Processing

Variables that are associated with vancomycin PK/PD were selected. These variables included the total dose of vancomycin from start to end, the usage of the loading dose at the first use, the total number of vancomycin infusions, the dose of vancomycin per infusion, the mean infusing interval of vancomycin, the interval from the start of vancomycin administration to the measurement of vancomycin serum levels, age, sex, height, body weight, serum creatinine levels, serum vancomycin levels, dialysis, and the total volume of transfusion from the start of vancomycin administration to the measurement of vancomycin levels. Patients with impaired kidney function may have issues with the metabolism and elimination of vancomycin. The estimated glomerular filtration rate (eGFR), which represents renal function, was calculated using the Chronic Kidney Disease Epidemiology Collaboration (CKD-EPI) formula and used as a variable. For the normalization of the data, for both the input and target variables, all values were scaled in the range (−1 to 1) using the minimum-maximum scaler. Moreover, we only had binary categorical variables in the model. Therefore, no special categorical variable encoding was performed in the model but a simple change to 0 and 1. To select significant input variables, we grouped variables into 5 categories. Each variable was classified as (1) baseline demographics—age, sex, body weight, and height; (2) drug administration-related variables; (3) variables related to the volume of distribution; (4) eGFRs using serum creatinine levels; and (5) variables related to the amount of transfusion. With the above-grouped categories, first, we have selected the default variables that would be used throughout all scenarios: the baseline demographics and the drug administration–related variables. Then, from the remaining 3 categories, with the clinician’s consultation, we tried to minimize the redundancy of the variables that would have the same meaning. Moreover, in accordance with the feature importance of each variable, we were able to find the best combination of model input variable sets. Furthermore, the contribution of the input data to the model in the test set was determined through the Shapley value of Shapley Additive exPlanations (SHAP) [19]. A high mean absolute estimated Shapley value indicates that the variable has a stronger impact on the output value.

### Statistical Analysis

The primary end point was the predictive performance of the vancomycin level within the therapeutic range. The baseline variables and patient characteristics of internal and external data sets were presented as frequencies with percentages or mean values with SDs. Between–data set comparisons were performed using the paired 2-tailed *t* test for continuous variables or the chi-square test for categorical variables. The measured serum vancomycin levels were used as true values. The predictive abilities of 4 kinds of models for vancomycin trough concentrations, using the PPK model on the website of the infectious diseases management program at the University of California, San Francisco [20], machine learning models (XGBoost) [21], and deep learning models (TabNet [22] and JointMLP), were evaluated for bias and precision by calculating the mean absolute error (MAE), root-mean-square error (RMSE), *R*^2^, and adjusted *R*^2^. The paired *t* test on MAE was used [23,24] to confirm significant differences in prediction performance between models. *P* values of <.05 were considered statistically significant. All analyses were performed with R software (version 3.4.4; R Foundation for Statistical Computing) and Python (version 3.8.12; The Python Software Foundation) using the *SciPy* 1.9.1, *PyTorch* 1.10.2, and *PyTorch Lightning* 1.6.4 libraries.

### Ethical Considerations

This study involved a retrospective analysis of medical records and did not involve collecting prospectively obtained data. As such, the study was considered to pose minimal risks and did not directly affect patients’ rights, welfare, or clinical care while offering recognized social benefits. To protect patient confidentiality during data collection and record review, all investigative records were securely stored in an encrypted database, and published results did not include any participants’ names or identifiers. The study received ethical approval from the institutional review board of Dongguk University Hospital (DUIH 2021-08-015) and Kangwon National University Hospital’s Institutional Review Board (KNUH-2022-03-020). This study was performed per the principles of the Declaration of Helsinki.

## Results

### Overview

Out of the 977 patients included in the DUIH data set, the testing data set was composed of 98 (10%) patients, with the other 879 patients belonging to the training data set. All 1429 patients included in KNUH were for external validation. The baseline characteristics did not differ significantly between the testing and training groups (Table 1). However, there were more older patients and shorter heights in the external validation set. The estimated renal function by the CKD-EPI method was worse in the patients for external validation. The patients included in MIMIC-IV showed significant differences in sex distribution compared to internal data sets or patients in other ICUs in Korea, and the proportion of non-Hispanic White patients was over 66.4% (n=1591).

**Table 1:** Baseline characteristics of all enrolled patients.

		Internal data set			External data set				External data set			
Variable		Test (n=98)	Train or validation (n=879)	From KNUH^a^ (n=1429)		*P* value^b^		From MIMIC-IV^c^ (n=2395)			*P* value^d^	
Female, n (%)		31 (31.6)	328 (37.3)	555 (38.8)		.32		1021 (42.6)			.002	
Age (years), mean (SD)		67.65 (15.49)	68.38 (15.56)	70.48 (14.39)		.002		65.38 (15.64)			<.001	
Height (cm), mean (SD)		163.56 (9.04)	162.42 (9.61)	161.36 (9.51)		.006		168.87 (10.67)			<.001	
Body weight (kg), mean (SD)		58.54 (15.80)	57.02 (13.27)	58.36 (13.60)		.06		83.01 (25.20)			<.001	
**Race, n (%)**							>.99					<.001
	Asian	98 (100)	979 (100)	1429 (100)				51 (2.1)				
	African-American	0 (0)	0 (0)	0 (0)				219 (9.1)				
	Hispanic	0 (0)	0 (0)	0 (0)				81 (3.4)				
	Non-Hispanic White	0 (0)	0 (0)	0 (0)				1591 (66.4)				
Serum trough value (mg/L), mean (SD)		14.49 (10.33)	15.01 (10.29)	13.37 (9.49)		<.001		17.58 (8.44)			<.001	
Serum creatinine (μmol/L), mean (SD)		1.10 (1.16)	1.11 (1.32)	1.06 (1.34)		.66		1.55 (1.34)			<.001	
Dialysis, mean (SD)		3 (3.1)	41 (4.7)	55 (3.8)		.55		144 (6.0)			.099	
The total dose of vancomycin before TDM^e^ (mg), mean (SD)		3961.28 (2812.91)	4289.52 (3504.59)	4446.94 (4933.77)		.45		3213.99 (2488.45)			<.001	
Number of vancomycin injections, n (%)		4.42 (2.72)	4.80 (4.03)	4.77 (5.17)		.75		3.10 (2.25)			<.001	
The average dose of vancomycin before TDM (mg), mean (SD)		882.40 (207.66)	891.90 (186.89)	931.31 (184.90)		<.001		1030.84 (171.60)			<.001	
Time between administration of vancomycin and the TDM (day), mean (SD)		3.81 (1.37)	4.01 (2.70)	2.86 (2.62)		<.001		2.54 (2.07)			<.001	
Interval among each dose of vancomycin (hours), mean (SD)		21.43 (11.59)	21.12 (11.75)	16.90 (10.58)		<.001		17.88 (12.06)			<.001	
Vancomycin loading, mean (SD)		8 (8.2)	54 (6.1)	0 (0.0)		<.001		0 (0.0)			<.001	
Volume distribution (L), mean (SD)		40.98 (11.06)	39.92 (9.29)	40.86 (9.52)		.06		58.11 (17.64)			<.001	
Adjusted volume distribution (L), mean (SD)		39.38 (3.81)	39.17 (3.34)	39.82 (3.25)		<.001		44.38 (5.74)			<.001	
CrCl^f^ (minutes), mean (SD)		91.49 (73.26)	84.06 (58.85)	90.36 (82.44)		.13		82.56 (64.02)			<.001	
CrCl (hours), mean (SD)		0.08 (0.06)	0.07 (0.05)	0.08 (0.07)		.13		0.07 (0.05)			<.001	
The elimination rate constant at infusion time (Kt), mean (SD)		1.70 (1.26)	1.59 (1.21)	1.17 (1.07)		<.001		1.07 (0.85)			<.001	
Trough levels of vancomycin (mcg/mL), mean (SD)		10.07 (9.15)	12.19 (14.62)	20.87 (20.79)		<.001		18.84 (22.81)			<.001	
eGFR^g^ MDRD^h^, mean (SD)		116.94 (100.14)	114.23 (90.33)	122.70 (123.49)		.20		73.85 (67.13)			<.001	
eGFR CKD-EPI^i^, mean (SD)		82.33 (39.87)	82.00 (38.47)	84.35 (36.96)		.33		62.94 (38.54)			<.001	
Vancomycin clearance, mean (SD)		4.00 (3.03)	3.69 (2.43)	3.96 (3.41)		.13		N/A			N/A	

^a^KNUH: Kangwon National University Hospital.

^b^*P* value: comparison of the internal data set with the external data set from KNU hospital in Korea.

^c^MIMIC-IV: Medical Information Mart for Intensive Care–IV.

^d^*P* value: comparison of the internal data set with the external data set from MIMIC-IV.

^e^TDM: therapeutic drug monitoring.

^f^CrCl: creatinine clearance.

^g^eGFR: estimated glomerular filtration rate.

^h^MDRD: Modification of Diet in Renal Disease.

^i^CKD-EPI: Chronic Kidney Disease Epidemiology Collaboration.

### XGBoost (1 of the Machine Learning Models) Versus PPK Model

Compared to the PPK model, the XGBoost model only significantly improved the predictive performance of external data sets (20.43 vs 11.59, a 76% change by RMSE and 11.87 vs 8.75, a 36% change by MAE; Table 2) when evaluated by RMSE and MAE. Although statistical significance could not be determined, the predictive power of internal data sets also improved by 8% (10.38 vs 9.58 by RMSE) and 16% (6.68 vs 5.75 by MAE).

**Table 2:** Performances of different models with bootstrapping.

Model	Internal, RMSE^a^ (95% CI)	External, RMSE (95% CI)	Internal, MAE^b^ (95% CI)	External, MAE (95% CI)	Internal, *R*^2^ (95% CI)	External, *R*^2^ (95% CI)
PPK^c^	10.38 (7.38 to 13.42)	20.43 (18.15 to 22.64)	6.68 (5.29 to 8.45)	11.87 (11.05 to 12.75)	−0.02 (−0.44 to 0.22)	−3.64 (−5.16 to −2.48)
XGBoost^d^	9.58 (6.31 to 12.6)	11.59 (10.88 to 12.17)	5.75 (4.37 to 7.48)	8.75 (8.34 to 9.13)	0.13 (−0.63 to 0.48)	−0.49 (−0.81 to −0.24)
TabNet	8.81 (6.33 to 11.29)	13.89 (11.01 to 17.71)	5.85 (4.53 to 7.25)	7.50 (6.90 to 8.13)	0.26 (−0.15 to 0.51)	−1.15 (−2.53 to −0.38)
300-layer MLP^e^	10.17 (7.06 to 13.09)	9.94 (8.84 to 11.04)	6.98 (5.61 to 8.63)	7.45 (6.55 to 7.28)	0.021 (–0.086 to 0.056)	–0.098 (–0.26 to –0.023)
JointMLP^f^ (proposed)	8.27 (5.33 to 11.19)	9.50 (8.72 to 10.30)	5.11 (3.92 to 6.58)	6.56 (6.18 to 6.90)	0.35 (−0.03 to 0.59)	−0.005 (−0.17 to 0.13)

^a^RMSE: root mean squared error.

^b^MAE: mean absolute error.

^c^PPK: population pharmacokinetic.

^d^XGBoost: extreme gradient boosting.

^e^MLP: multilayer perceptron.

^f^JointMLP: joint multilayer perceptron.

The TabNet model showed significantly better predictive performance for vancomycin TDM after vancomycin treatment than the PPK model in external data sets (20.43 vs 13.89, a 47% change by RMSE and 11.87 vs 7.50, a 58% change by MAE; *P*<.001, respectively). Contrary to the prediction improvements compared to the PPK model in the external data set (TabNet 47% vs XGBoost 76% by RMSE), there was no significant difference between TabNet and XGBoost in predictive power for vancomycin TDM on the internal data set.

### JointMLP Versus Other Models

In both internal and external data sets, the JointMLP model had better predictive performance for vancomycin TDM among the models we compared. It improved the predictive power up to 31% (6.68 vs 5.11) based on MAE for the vancomycin concentration after vancomycin infusion on the internal data set compared to PPK. Additionally, our JointMLP model was more accurate (11.87 vs 6.56, 81%) in predicting the vancomycin levels on the external data set than the PPK method. The proposed JointMLP model significantly outperformed XGBoost and TabNet with a 13% (5.75 vs 5.11) and 14% (5.85 vs 5.11) improvement in predictive accuracy under MAE on the internal data set, respectively. Additionally, our trained JointMLP showed better performance not only on the internal data set but also on the external data set that consisted of 1429 patients with critical illnesses by 34% (8.75 vs 6.56) and 14% (7.50 vs 6.56) when compared with XGBoost and TabNet. Furthermore, JointMLP’s RMSE performance is higher than that of other models on all data sets, which means that the proposed model is more robust than theirs against outlier data.

**Figure 2:**
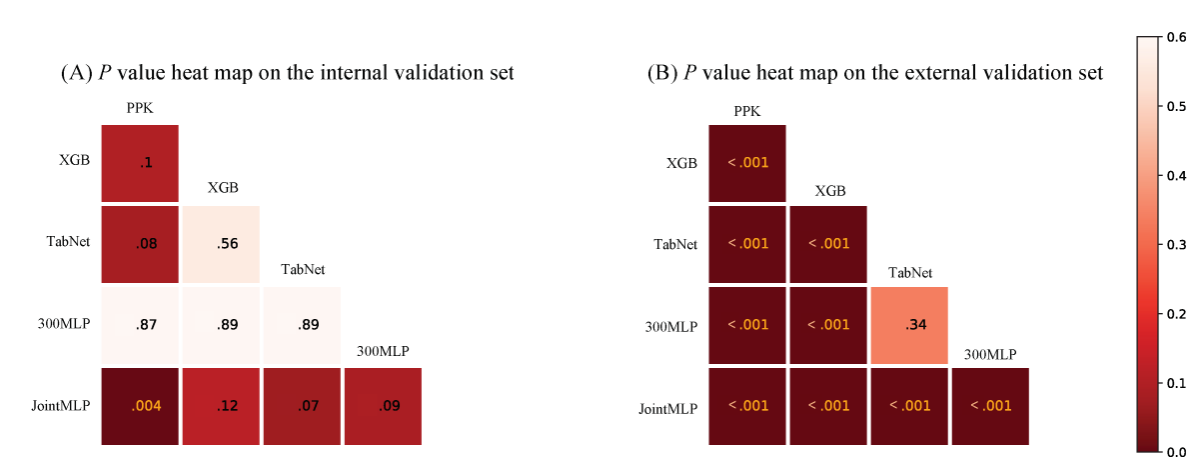
*P* value heat map by paired t test on all the combinations of prediction models. The *P* value for each comparison of the mean absolute error for predictive performance on (A) the internal validation data set (B) and the external validation data set. 300MLP: 300-layer multilayer perceptron; JointMLP: joint multilayer perceptron; PPK: population pharmacokinetic; XGB: extreme gradient boosting.

**Figure 3:**
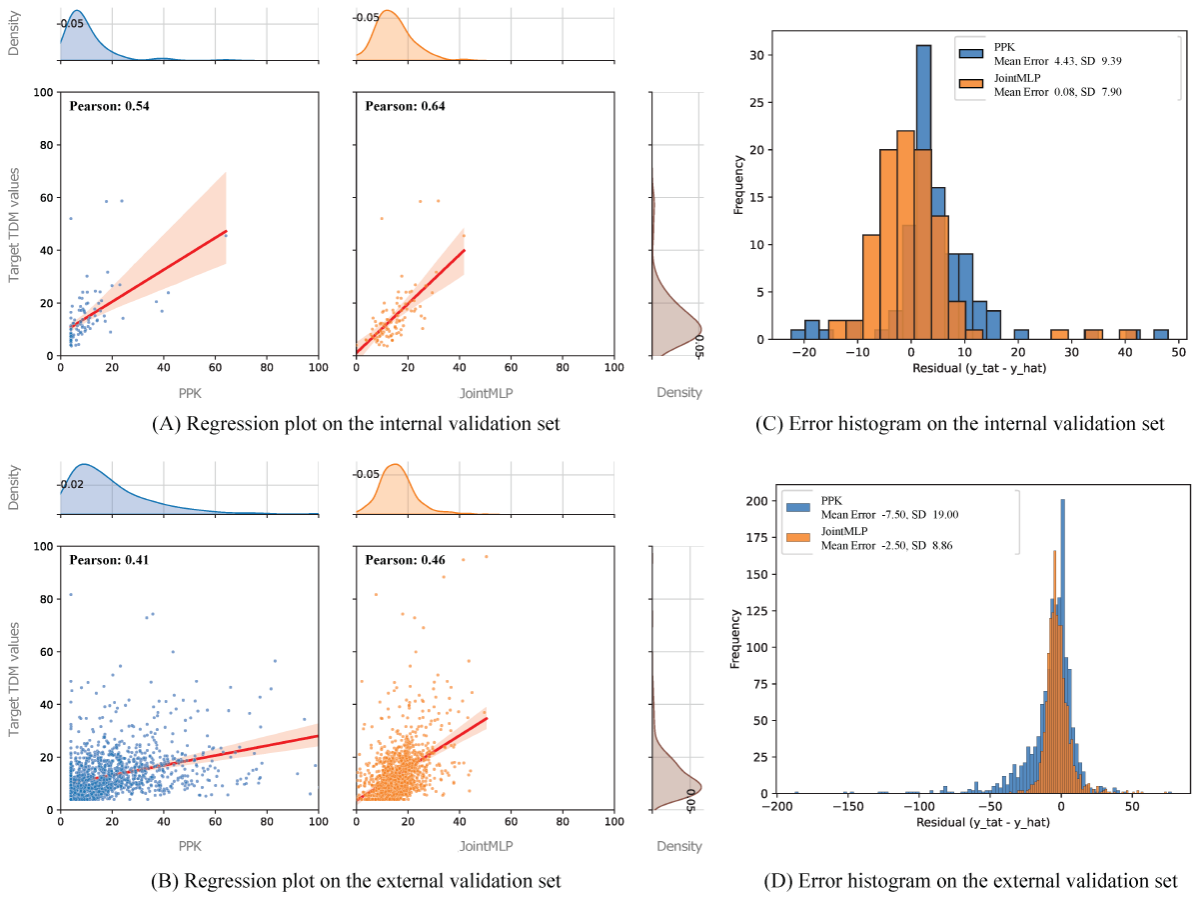
Comparisons of the population pharmacokinetic (PPK) and joint multilayer perceptron (JointMLP) models on the internal and external validation sets. Scatter plots of (A) the internal data set and (B) the external data set with the predicted value against a target value. The error histograms of (C) the internal data set and (D) the external data set with a predicted value against a target value. TDM: therapeutic drug monitoring.

Within the external data set, we clearly see that all models are statistically significant among themselves (Figure 2). However, the *P* value of JointMLP within the internal data set expressed a trend more robust than all other models while showing statistical significance only when compared with PPK with MAE. The mean error and variance of the JointMLP model were nearer to zero and smaller in both internal and external data sets than in the PPK model (Figure 3).

The most influential variables in TDM prediction were eGFR CKD-EPI, the average dose of vancomycin, and the average volume of distribution in both internal and external data sets. These variables consistently had high SHA*P* values in both data sets (Table 3).

**Table 3:** The mean values of Shapley Additive exPlanations on the internal and external validation sets.

Variables	Internal validation, mean (SD)	External validation, mean (SD)
eGFR^a^ CKD-EPI^b^	0.076 (0.09)	0.063 (0.088)
Average dose of vancomycin	0.045 (0.05)	0.04 (0.073)
Average volume of distribution	0.037 (0.029)	0.032 (0.04)
Interval of vancomycin	0.028 (0.014)	0.031 (0.03)
Body weight	0.027 (0.038)	0.024 (0.044)
Total dose	0.014 (0.028)	0.02 (0.066)
Height	0.013 (0.023)	0.015 (0.026)
Sex	0.01 (0.018)	0.011 (0.004)
Number of vancomycin injection	0.01 (0.012)	0.015 (0.031)
Creatinine	0.01 (0.007)	0.01 (0.011)
Loading	0.007 (0.012)	0.003 (0.01)
Age	0.006 (0.006)	0.006 (0.003)
Dialysis	0.005 (0.018)	0.005 (0.028)
The elimination rate constant at infusion time	0.005 (0.022)	0.005 (0.022)
Time between vancomycin injection and TDM^c^	0.003 (0.002)	0.007 (0.007)

^a^eGFR: estimated glomerular filtration rate.

^b^CKD-EPI: Chronic Kidney Disease Epidemiology Collaboration.

^c^TDM: therapeutic drug monitoring.

### JointMLP Versus 300-Layer MLP

The purpose of Table 2 was to compare the predictive performances of the 300-layer MLP model and the proposed JointMLP model. The results showed that JointMLP outperformed the 300-layer MLP model in both the internal (8.27 vs 9.94, a 23% improvement by RMSE, and 6.98 vs 5.11, a 37% improvement by MAE) and external (9.50 vs 9.94, a 5% improvement by RMSE, and 7.45 vs 6.56, a 14% improvement by MAE) data sets, indicating the effectiveness of the boosting ensemble approach used in JointMLP. Figure 2 shows the *P* value analysis of the comparison between JointMLP and the 300-layer MLP model. The analysis revealed that the difference between the 2 models was statistically significant (*P*<.001) within the external data set. However, compared to other models, the difference between JointMLP and the 300-layer MLP model was not statistically significant (*P*=.09) within the internal data set.

### Model Comparisons in the MIMIC-IV Data Set

The findings presented in Table 4 clearly indicated that the JointMLP model performed better than both the PPK and 300-layer MLP models by a significant margin of 160% and 25%, respectively, when evaluated using the RMSE metric. This improvement in predictive performance was observed even in the MIMIC-IV data set, which included patients from multiple centers and races. The JointMLP model also demonstrated superior performance compared to the XGBoost and TabNet models, with improvements of 5% and 4%, respectively. When examining the MAE metric, the JointMLP model significantly outperformed the PPK model by 90%, with further improvements of 8%, 6%, and 5% over the XGBoost, 300-layer MLP, and TabNet models, respectively.

**Table 4:** Performances of models with Medical Information Mart for Intensive Care–IV data set.

Model	MIMIC^a^, RMSE^b^ (95% CI)	MIMIC, MAE^c^ (95% CI)	MIMIC, *R*^2^ (95% CI)
PPK^d^	22.22 (18.88 to 25.72)	12.13 (11.42 to 12.87)	−5.93 (−8.52 to –3.99)
XGBoost^e^	8.93 (8.60 to 9.23)	6.93 (6.69 to 7.15)	–0.12 (−0.21 to 0.04)
TabNet	8.91 (8.53 to 9.25)	6.69 (6.45 to 6.93)	–0.11 (−0.17 to –0.06)
300-layer MLP^f^	10.65 (8.88 to 12.72)	6.80 (6.48 to 7.13)	–0.59 (–1.30 to –0.12)
JointMLP^g^ (proposed)	8.53 (8.17 to 8.88)	6.40 (6.17 to 6.64)	–0.02 (−0.08 to 0.03)

^a^MIMIC: Medical Information Mart for Intensive Care.

^b^RMSE: root mean squared error.

^c^MAE: mean absolute error.

^d^PPK: population pharmacokinetic.

^e^XGBoost: extreme gradient boosting.

^f^MLP: multilayer perceptron.

^g^JointMLP: joint multilayer perceptron.

**Figure 4:**
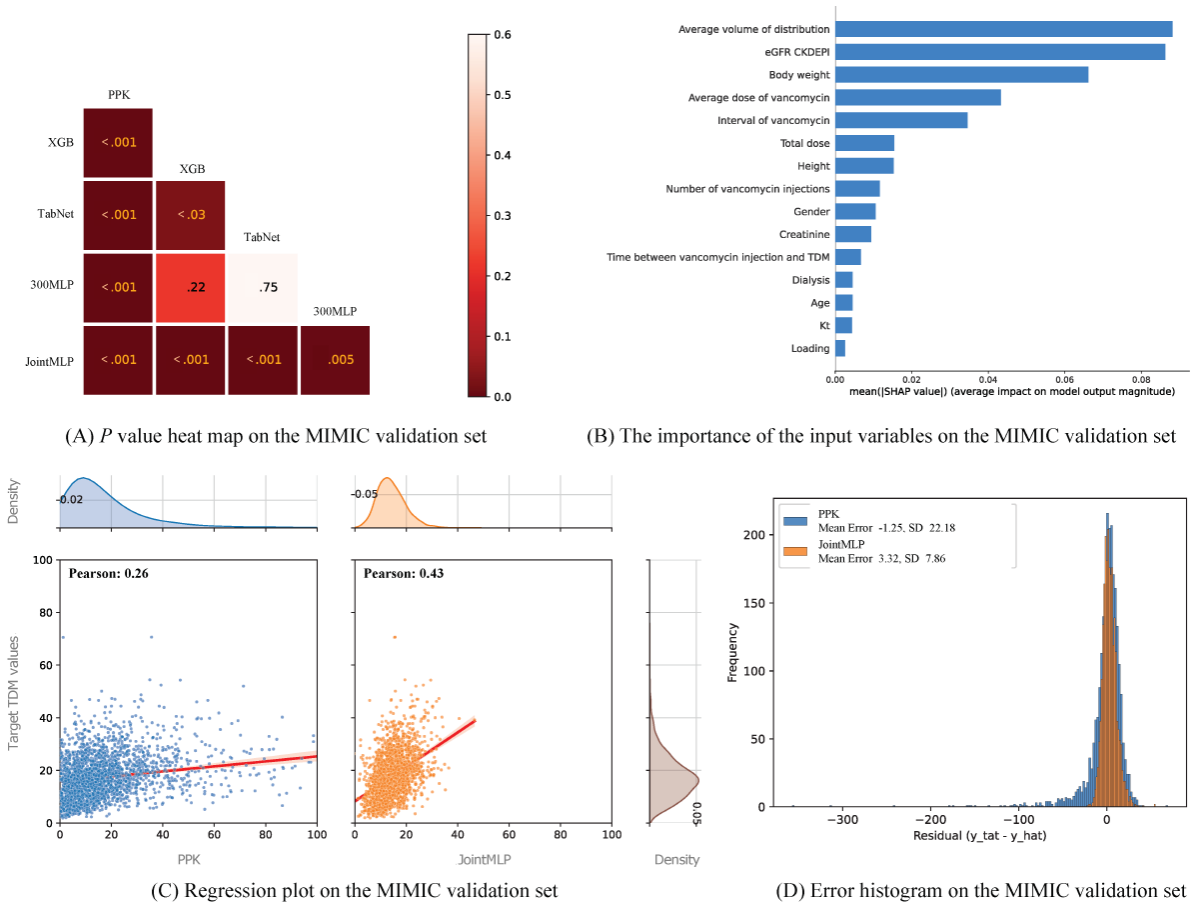
Result figures from the Medical Information Mart for Intensive Care (MIMIC) validation set. (A) The *P* value for each comparison of the mean absolute error for predictive performance on the MIMIC validation data set; (B) the Shapley Additive exPlanations (SHAP) values of the joint multilayer perceptron (JointMLP) model on the MIMIC validation set; (C) scatter plots of the MIMIC data set with the JointMLP predicted values against target values; and (D) the error histograms of the MIMIC data set with the JointMLP predicted values against target values. 300MLP: 300-layer multilayer perceptron; CKD-EPI: Chronic Kidney Disease Epidemiology Collaboration; eGFR: estimated glomerular filtration rate; PPK: population pharmacokinetic; TDM: therapeutic drug monitoring; XGB: extreme gradient boosting.

All models, except for the 300-layer MLP, were statistically significant in the MIMIC-IV data set (Figure 4A). The most influential variables in TDM prediction using the JointMLP model were consistent with eGFR CKD-EPI, average vancomycin dose, and average distribution volume, which were variables identified in the internal data set (Figure 4B). The 300-layer MLP was statistically significant (*P*<.001) compared to the PPK model. Additionally, the JointMLP model had a mean error and variance that were close to zero and smaller than those of the PPK model in the MIMIC-IV data set (Figure 4C and D).

## Discussion

### Principal Findings

The PPK method is widely used for predicting vancomycin TDM levels. However, it is population-dependent, leading to inappropriate results for some patients. Therefore, this study proposes a consistent decision-making system that is population-independent and unaffected by clinicians’ abilities by applying a joint MLP model that integrates various successful deep learning models and decision trees. The proposed JointMLP showed the best performance for predicting vancomycin trough concentration in all data sets compared to other models. Significantly, in the MAE metric, the JointMLP model improved performance by 90% over the PPK model. Results mean that the proposed model can be applied to predict appropriate vancomycin trough concentrations in patients with critical illnesses who require vancomycin treatment in various situations.

### Comparison to Machine Learning Model

In our study, we compared the performance of 3 machine learning models for predicting vancomycin levels: JointMLP, XGBoost, and TabNet. XGBoost is a popular gradient-boosting algorithm known for its high accuracy and flexibility, while TabNet is a newer deep-learning model [22] specifically designed for tabular data. Compared to XGBoost and TabNet, our JointMLP model outperformed both in terms of predictive accuracy. One of the main reasons for this is that our model was designed to handle noisy data samples, a common characteristic of medical data sets. A deep neural network architecture accomplishes this with multiple layers, which allows the model to learn complex relationships between the input features and the output variable. In contrast, XGBoost and TabNet are both tree-based models, which may need to be more effective at handling noisy data.

Compared to previous studies [15,24-26], our proposed JointMLP model performs better in accurately predicting vancomycin levels. Specifically, our model outperforms XGBoost and TabNet, 2 well-known models in analyzing tabular data and predicting drug concentrations. Moreover, our JointMLP model is designed to handle noisy data samples effectively, a crucial feature for real-world applications in the medical domain where data quality can be highly variable. Our model’s robustness is demonstrated by comparing it to a 300-layer MLP, confirming the effectiveness of our model structure in handling vancomycin TDM predictions. Importantly, our model is trained on a larger and more diverse data set than previous studies and tested on an unseen data set, demonstrating its generalizability and suitability for predicting drug concentrations. Overall, our findings highlight the potential of deep learning models in improving the accuracy and reliability of drug concentration prediction, which can lead to better treatment outcomes and improved patient health. Overall, our findings suggest that the JointMLP model is a promising approach for predicting vancomycin levels, particularly in the presence of noisy data. However, the choice of which model to use may depend on the specific characteristics of the data set being analyzed and the goals of the analysis.

### Limitations and Future Work

When considering implementing our approach in clinical settings, there are several factors to consider. First, although we extended our model to validate across diverse ethnic groups by including patients from Asia and other regions in the MIMIC-IV data set, we acknowledge that there is still room for improvement in this regard. We used a larger data set than previous studies, with 977 samples as the internal data set and 1429 samples as the external data set. Additionally, we sought to develop a generalized model by applying the MIMIC-IV data set, which includes 2394 samples. However, additional validation with external data sets is necessary to enhance further the generalizability of our model for predicting vancomycin levels. Second, our comparison of the predictive performance with the PPK method was based on the trough-based target method, which is easily used in the treatment of patients with critical illnesses, rather than the AUC/MIC-specific range that is currently recommended. Therefore, further research is needed to compare the AUC/MIC range with our approach.

Despite these limitations, our deep learning model, if implemented in actual clinical practice, could significantly improve the treatment outcomes of ICUs by supporting clinical decision-making in a more standardized and consistent manner. Our proposed JointMLP model will continue to evolve and update its performance to mimic the human brain better and determine the optimal vancomycin dose.

### Conclusions

In this study, proposing the JointMLP approach in clinical settings has significant implications for public health, as it can help optimize treatment outcomes for patients with critical illnesses in ICUs. By providing a more accurate and consistent method for predicting vancomycin levels, the model can reduce adverse events associated with suboptimal vancomycin dosings, such as the increased risk of bacterial resistance, longer hospital stays, and higher health care costs. Furthermore, the ongoing development and improvement of the JointMLP model through continuous learning and updating can lead to more effective treatments and better health outcomes for patients, improving the accuracy and reliability of other clinical prediction models in the future. Overall, the implementation of the JointMLP approach has the potential to improve public health outcomes and benefit patients worldwide.

## Abbreviations

AUC: area under the curve
CKD-EPI: Chronic Kidney Disease Epidemiology Collaboration
DUIH: Dongguk University Ilsan Hospital
eGFR: estimated glomerular filtration rate
ICU: intensive care unit
JointMLP: joint multilayer perceptron
KNUH: Kangwon National University Hospital
MAE: mean absolute error
MIC: minimum inhibitory concentration
MIMIC-IV: Medical Information Mart for Intensive Care–IV
MLP: multilayer perceptron
PK/PD: pharmacokinetic/pharmacodynamic
PPK: population pharmacokinetic
RMSE: root-mean-square error
SHAP: Shapley Additive exPlanations
TDM: therapeutic drug monitoring
XGBoost: extreme gradient boosting

## Data Availability

The data used and analyzed during this study are available from the corresponding author on reasonable request.
